# Performance of the Xpert HPV assay in women attending for cervical screening

**DOI:** 10.1016/j.pvr.2015.05.002

**Published:** 2015-06-16

**Authors:** Jack Cuzick, K. Cuschieri, K. Denton, M. Hopkins, M.A. Thorat, C. Wright, H. Cubie, C. Moore, M. Kleeman, J. Austin, L. Ashdown-Barr, K. Hunt, L. Cadman

**Affiliations:** a Centre for Cancer Prevention, Wolfson Institute of Preventive Medicine, Charterhouse Square, London, EC1M 6BQ, United Kingdom; b Scottish Human Papillomavirus Reference Laboratory, Royal Infirmary of Edinburgh, 51 Little France Crescent, Edinburgh EH16 4SA, United Kingdom; c Severn Pathology, Pathology Sciences Building, Southmead Hospital, Westbury-on-Trym, Bristol, BS10 5NB, United Kingdom; d Specialist Virology Centre at Liverpool Clinical Laboratories, Royal Liverpool University Hospital, Royal Liverpool University Hospital, Prescot Street, Liverpool L7 8XP, United Kingdom; e Pathology Department, Mint Wing, Imperial College Healthcare NHS Trust, St Mary׳s Hospital, Praed Street, Paddington, London W2 1NY, United Kingdom

**Keywords:** Human papillomavirus, Xpert, Cervical screening, HPV testing

## Abstract

**Objectives:**

This study evaluated the Xpert HPV Assay in women attending screening in general practice by comparing Xpert with two established HPV tests, cytology and histology.

**Methods:**

A prospective study in women aged 20–60 years attending screening in Bristol, Edinburgh and London using residual Preservcyt cytology samples. Sample order was randomised between Roche cobas4800 and Cepheid Xpert assays with Qiagen hc2 third.

**Results:**

3408 cases were included in the primary analysis. Positivity for Xpert was 19.6%, cobas 19.2% and hc2 19.9% with high concordance (kappa=86.8% vs cobas, 81.55 vs hc2). Xpert, cobas and hc2 showed similar sensitivity (98.7%, 97.5%, 98.7%) for CIN2+. All pairwise comparisons had high concordance (Kappa ≥0.78 with any abnormal cytology. Xpert and hc2 were positive for all cases of ≥moderate dyskaryosis (*N*=63)), cobas was negative in two. Histology was available for 172 participants. 79 reported CIN2+, 47 CIN3+. All CIN3+ was positive on Xpert and hc2 and one case negative for cobas. One case of CIN2 was negative for all assays.

**Conclusions:**

The performance of Xpert HPV Assay in a general screening population is comparable to established HPV tests. It offers simplicity of testing, flexibility with non-batching of individual samples and rapid turnaround time.

## Highlights

• This is the first study to report on the performance of the Xpert HPV Assay in a screening population.
• Xpert HPV Assay׳s performance in detecting HPV is comparable to two clinically validated HPV tests.
• Xpert HPV Assay showed a relative sensitivity of 98.73% for CIN 2 or higher and 100% for CIN3 or higher.

## 1 Introduction

Human Papillomavirus (HPV) testing is increasingly being recognised as the best method for primary cervical screening because of its very high sensitivity [1], [2], [3]. There are a number of HPV tests currently available, but they all require a high degree of laboratory expertise and sophisticated platforms which are generally more efficient when samples are run as full batches. The Xpert HPV Assay (Cepheid, Inc, Sunnyvale, CA) is a real-time Polymerase Chain Reaction (PCR) assay, using disposable cartridges, for the detection of 14 types of high-risk HPV DNA (types 16, 18, 31, 33, 35, 39, 45, 51, 52, 56, 58, 59, 66, 68) from a liquid cytology sample. The assay is run on the Cepheid GeneXpert System which is a scalable, random access instrument platform. Its capacity ranges from one to 80 tests in simultaneous, but asynchronous assays which can be run for the same or different assays according to the cartridge used. Results are typically available in 60–90 min depending on the assay. An early and now widespread use of the platform was to diagnose tuberculosis and distinguish drug resistant strains in low and intermediate income countries [4]. Other uses include testing for a range of infectious agents. There are now over 8500 platforms in use throughout the world.

To date, performance of the Xpert HPV Assay using samples derived from referral populations have been comparable to those of currently available clinically validated tests [5], [6]. The objective of this study was an evaluation of the Xpert HPV Assay in a screening population using residual cervical cytology PreservCyt specimens originally obtained from women aged 20–60 years. Performance of the Xpert HPV Assay was compared to two established HPV DNA tests and with cytology and histopathology.

## 2 Methods

There were four sites in this study: 3 clinical and also 1 reference. The external reference site gave blind testing and standardised conditions for the primary comparator – cobas 4800. The three clinical sites were Bristol, Edinburgh and London. Samples were originally collected from women attending for routine cervical screening as part of the UK NHSCSP in PreservCyt liquid cytology using a Cervex brush (Rovers Medical Devices B.V., Oss, Netherlands) or similar. A residual specimen was the remaining PreservCyt sample, kept at room temperature, after the completion of routine clinical testing (cytology evaluation and, where applicable, high-risk HPV triage). A satisfactory cytology result (with or without HPV triage) and sufficient remaining sample volume to carry out at least two HPV tests (minimum 8 ml PreservCyt) was required. Sample size calculations were done separately for women aged 20–29 years and aged 30–60. An HPV prevalence of 25% in the younger women and 8.7% in the older age group resulted in minimal sample sizes of 900 and 2600 respectively to be able to have 95% power to ensure that the lower 2-sided 95% CI for kappa would be above 0.75 when the true value was 0.82 for cobas and above 0.65 when the true value was 0.75 for hc2 for each age group. Assumed true kappa values were based on previous studies [7], [8].

The samples were collected from women who had originally attended screening between July 2013 and January 2014. Populations were somewhat different as the screening algorithms were different in the 3 centres leading to different positivity rates. In order to obtain 900 women in the 20–29 age group, this was oversampled in Edinburgh, as screening begins at age 20 years in Scotland and 25 years in England. Also in Scotland women who had borderline or mildly dyskaryotic smears were followed by repeat short term cytology and not triaged with HPV to determine if they should be referred immediately, so that these women were also included in the sample from Edinburgh. However within each centre samples were collected at random from each age stratum.

The primary comparison was between the Xpert HPV Assay and the Roche cobas 4800 HPV test (Roche Diagnostics, Basel, Switzerland) with a secondary comparison between Xpert HPV Assay and the hc2 test (QIAGEN, Gaithersberg, MD). Aliquot order was either by method 1, where odd numbered specimens had an aliquot for cobas detection taken first and Xpert second and vice versa for even numbers or method 2 where batches of 100 specimens were divided into 2 halves with the first 50 having an aliquot for cobas detection taken first and Xpert second and the next 50 having the Xpert first and cobas second. The aliquot for hc2 was always taken third. The original specimen in the ThinPrep pot was mixed either by inverting 8–10 times or briefly vortexing for 5–10 s prior to the removal of each aliquot. The cobas aliquot (volume 2 ml) was stored at room temperature (15–25 °C) and dispatched to the reference laboratory in Liverpool where testing was conducted in accordance with the manufacturer׳s instructions. The Xpert HPV Assay was conducted onsite in laboratories in Bristol, Edinburgh and London. The aliquot (volume 1.2 ml) was stored at room temperature (15–25 °C) prior to testing. Testing was carried out within six weeks of sample collection in accordance with manufacturer׳s instructions. The aliquot for hc2 testing (volume 4 ml or 2 ml depending upon whether a manual or Rapid Capture System processing method was used) was stored at room temperature or refrigerated (2–30 °C) and tested onsite within six weeks, as per manufacturer׳s instructions.

Detailed technical descriptions of the Xpert HPV Assay have been reported elsewhere [5]. Briefly, the E6 and E7 genes of the 14 targeted HR HPV types are amplified simultaneously in five fluorescent channels: HPV16; HPV18/45; HPV31/33/35/52/58; HPV51/59; and HPV39/56/66/68. A specimen adequacy control, HMBS, is detected in a sixth channel. Assay results were reported as an overall “positive” if any of these types were detected, with HPV16 and HPV 18/45 reported separately.

The cut-off for positivity with the Xpert HPV Assay was<40Ct for HPV 16 and HPV 18/45, and <38 Ct for HPV 31/35/33/52/58, HPV 51/59, and HPV 39/68/56/66. Positivity for Cobas was <40Ct for HPV 31/33/35/39/45/51/52/56/58/59/66/68 and HPV16 and <40.5 Ct for HPV 18 and>1 RLU for hc2.

Positivity rates and agreement of the Xpert Assay with the Cobas (primary comparator) and high-risk hc2 (secondary comparator) were evaluated. Referral to colposcopy was only based on the cytology result, with or without HPV triage according to local protocols. The triage tests used were Abbott RealTime in London and hc2 and Aptima in Bristol. Scotland, at the time of the study, started routine cervical screening at age 20 years whereas in England it was age 25 years. The Edinburgh centre therefore had a younger age distribution. Scotland is due to change to 25 years in 2016. Triage also differed between the two countries. In England, but not Scotland, borderline squamous or mildly dyskaryotic cytology is followed by HPV triage testing. Those women testing negative are followed up with repeat cytology whereas those testing positive are referred for colposcopic examination, where histopathology samples may be taken for further assessment. In Scotland there is currently no HPV triage and women are referred for colposcopy only after more than one consecutive low grade cytology result. Women with higher grade cytology results were referred for colposcopic assessment in all centres [9], [10]. Histology results for the English sites were collected up to 12 months after cytology but this was extended to 18 months for Edinburgh in view of the different referral guidelines. The HPV results from this study were not used for patient management, so that lesions in women with negative cytology could not be identified.

Ethics approval was received from National Research Ethics Service Committee East Midlands – Leicester (reference: 13/EM/0244) on 10th June 2013. Individual informed consent was not required as the study was non-invasive and the samples would otherwise have been discarded. In addition the residual samples were pseudo-anonymised and only identifiable to the research team by subject number. Cytology data and histopathology data were linked to the HPV result by the centre and then all data were pseudo-anonymised before release to the statistical centre and were only identifiable by subject number.

## 3 Results

**Fig. A1:**
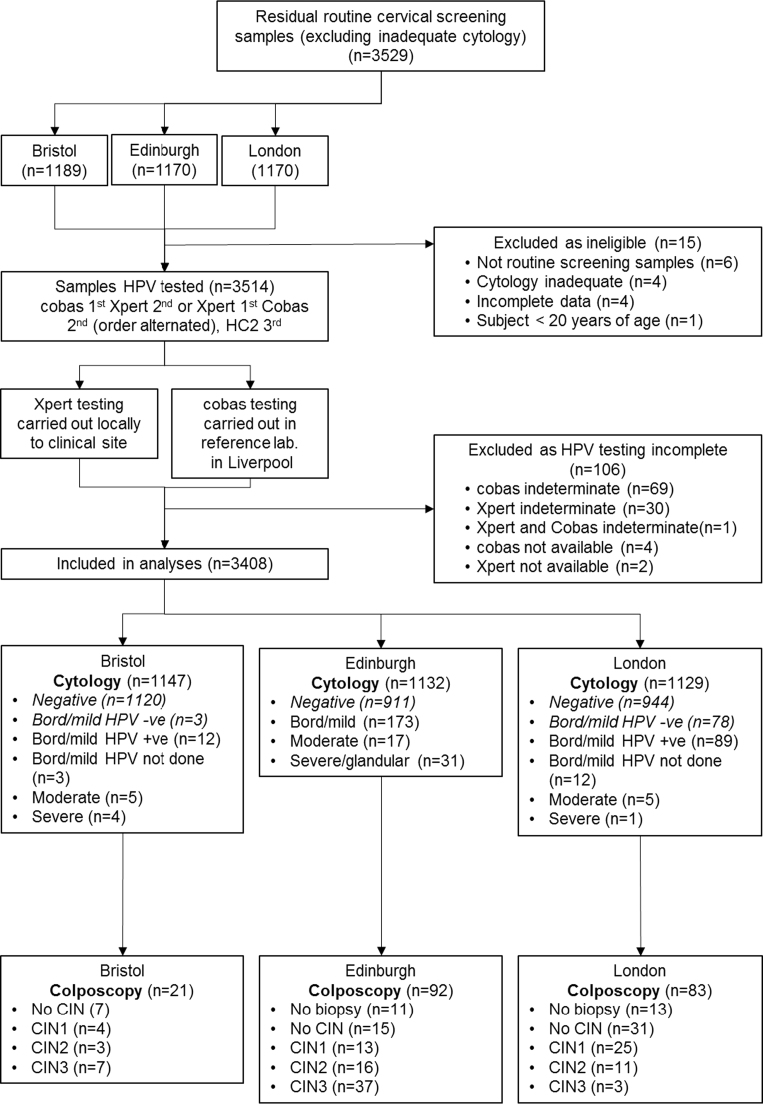
Flowchart of women in this screening study.

A total of 3529 samples were collected. Of these 15 women were found to be ineligible and another 106 samples did not produce a valid result for both Xpert and cobas leaving 3408 women in the primary analyses (Fig. A1) with all three tests valid, no hc2 tests were invalid. Their demographic and cytology details are shown in Table B1. The median age was 38 years and 76% were aged 30 years or older, but the Edinburgh cohort was younger due a policy of screening from age 20 years. The negative cytology rate of 87% overall ranged from 98% in Bristol to 80% in Edinburgh and 84% in London. 63 (2%) women had moderate or severe dyskaryosis on cytology with Edinburgh accounting for 48 of these. A further 370 (11%) women overall had borderline or mild dyskaryosis. Bristol had a very low rate of low grade abnormality (2% compared to 15% in Edinburgh and 16% in London). Positivity by test, age group, and cytology and histology outcome is shown in Table C1. 19.6% of all women were positive by Xpert HPV Assay compared with 19.2% with cobas and 19.9% with hc2. Supplementary Table 1 shows that there was variation of positivity rates by centre reflecting the differences above in positivity in cytology, with Bristol reporting 12% positive with Xpert, 12% with Cobas and 12% with hc2 compared to Edinburgh 25%, 25% and 26% respectively and London 21%, 21% and 22% respectively.

Table C1 also shows that Xpert and hc2 were positive for all cases of high grade cytology whereas cobas was negative for 2 cases, in women aged 29 years. Xpert and hc2 were also positive for all cases of CIN3+, whereas cobas was negative for one women aged 29 (who was one of the women with abnormal cytology). In addition a 31 year old woman with CIN2 on excisional biopsy was negative for each HPV test. Her original cytology result was reported as low grade but she had a subsequent moderate result and her excision histology result was 11 months after her sample for HPV testing was taken.

Concordant and discordant outcomes between Xpert and the other two tests are shown in Table D1. High levels of agreement in HPV positivity were seen between Xpert and the other two tests (95.8% for cobas and 94.1% for hc2). Discordant pairs were of similar number in each direction reflecting similar overall positivity rates. All tests were positive for all high grade cytology and almost all CIN2+. Cobas was negative for two cases of high grade cytology. One subsequently had CIN3 on excision biopsy whereas the other had no CIN on histology. All three tests showed similar specificity for CIN2+ (82–83%) and CIN3+ (81–82%) and also similar positivity predictive values. However both of these were age dependent with specificity being substantially higher in women aged 30–60, but PPV higher in women aged 20–29 years than for women aged 30–60 years. Negative predictive values were all above 99.8% (data not shown). Supplementary Table 2 breaks these numbers down by site.

HPV 16 was reported separately for the Xpert and cobas assays. For Xpert positivity rates for HPV16 were 5% overall and 14% for women aged 21–29 and 2% for ages 30–60. For cobas the corresponding rates were the same 5%, 14% and 2%. Table D1 shows the concordance rate was very high being 99.0% with a kappa value of 90.1% and similar numbers of discordants in each direction.

## 4 Discussion

This is the first study to assess the performance of the Xpert HPV Assay in a screening population. The data suggest that the performance of Xpert is highly comparable to two clinically validated tests (cobas 4800 and hc2). Xpert HPV Assay offers more extensive partial typing than cobas which reports HPV 16 and HPV 18 and a consensus positivity for 12 other high risk HPV types. Hc2 does not offer individual typing but a consensus result for 13 high risk types. The Xpert HPV Assay also has the advantage that results are available within a short timeframe (the assay run time is under 1 h) with the flexibility to run a single or multiple cartridges. The system is very accessible operationally, requiring simple vortexing of the ThinPrep pot and pipetting into the single cell cartridge which is then slotted into the GeneXpert machine. This flexibility with non-batching and rapid turnaround time of individual samples could prove particularly useful in laboratory and clinical settings where workload is variable.

The sensitivity and specificity results in this study were similar across centres, despite their different disease rates. However these are relative measures since colposcopy referral was based only on routine procedures and so some disease in cytology negative women may have been missed.

There were differences between centres in the proportion of abnormal histology and cytology and HPV prevalence. There are a number of possible explanations for this. Women under 25 years were targeted in Edinburgh whereas there were very few in London and Bristol where screening commences at 25 years, although invitations can be sent six months earlier. Subsequently there were differences between centres in the age distribution. In addition there were different referral policies between England and Scotland. For example Bristol has incorporated HPV triage as part of screening for substantially longer than London, and this was not used at all in Edinburgh at the time of this study so that those from Edinburgh with up to two low risk cytology results were kept within the ‘screening’ population. The higher HPV and disease rate in Edinburgh had the advantage or providing more CIN2+ cases overall which increased the accuracy of the sensitivity estimates. However, no differences between the performance of the three HPV tests was seen across the different centres. Overall the HPV prevalence was only slightly higher here than that previously recorded in a similar English population as part of the ARTISTIC Study (19.9% versus 16.1% using hc2), but ARTISTIC used a 2RLU cut-off for hc2. Cytology results were also virtually identical with 10.9% low grade and 1.9% high grade abnormalities for both studies [11]. Lastly histology was assessed locally without central review, so there could be local differences in interpretation across centres.

In conclusion, this study shows that the Xpert HPV Assay is a useful additional to the HPV testing market and compares well in terms of performance with other more established HPV testing systems. A study in which the HPV result is used for clinical management is needed to demonstrate the added sensitivity of this test in cytology negative women and directly compare its performance to cytology.

## Figures and Tables

**Table B1:** Demographics and cytology results.

	**Bristol**	**Edinburgh**	**London**	**All**
	***N*****(% site total)**	***N*****(% site total)**	***N*****(% site total)**	
**Number included**	1147	1132	1129	3408
**Age**				
Median (IQR)	41 (32–49)	38 (29–48)	35 (28–44)	38 (30–47)
≥30y	953 (83.1)	844 (74.6)	784 (69.4)	2581 (75.7)
**Cytology**				
Negative	1120 (97.6)	911 (80.5)	944 (83.6)	2975 (87.3)
Borderline or mild	18 (1.6)	173 (15.3)	179 (15.8)	370 (10.9)
High grade or glandular	9 (0.8)	48 (4.2)	6 (0.5)	63 (1.8)

**Table C1:** HPV positivity by test, age group, cytology and histology result.

**Age (y)**	**Cytology**				**Histology**	
	*N* (% of all with same cytology and age)				*N* (% of all with same histology and age)	
Total	Normal	Low grade	High grade	All	CIN2+	CIN3+
20–29	604	180	43	827	52	33
30–60	2371	190	20	2581	27	14
All	2975	370	63	3408	79	47
**Xpert +ve**						
20–29	160 (26.5)	133 (73.9)	43 (100)	336 (40.6)	52 (100)	33 (100)
30–60	214 (9.0)	98 (51.6)	20 (100)	332 (12.9)	26 (96.3)	14 (100)
All	374 (12.6)	231 (62.4)	63 (100)	668 (19.6)	78 (98.7)	47 (100)
**Cobas +ve**						
20–29	163 (27.0)	132 (73.3)	41 (95.3)	336 (40.6)	51 (98.1)	32 (97.0)
30–60	199 (8.4)	98 (51.6)	20 (100)	317 (12.3)	26 (96.3)	14 (100)
All	362 (12.2)	230 (62.2)	61 (96.8)	653 (19.2)	77 (97.5)	46 (97.9)
**hc2 +ve**						
20–29	143 (23.7)	135 (75.0)	43 (100)	321 (38.8)	52 (100)	33 (100)
30–60	224 (9.4)	113 (59.5)	20 (100)	357 (13.8)	26 (96.3)	14 (100)
All	367 (12.3)	248 (67.0)	63 (100)	678 (19.9)	78 (98.7)	47 (100)

**Table D1:** Discordant results according to test – overall and according to cytology and histology categories. Differences for HPV16 between Xpert and cobas shown as final column.

**Population/Concordance**	**Xpert vs Cobas**	**Xpert vs hc2**	**Cobas vs hc2**	**Xpert vs Cobas HPV 16**
**All –*****N*****=3408**				
Agreement (%)	95.8	94.1	93.7	99.0
Kappa (95%CI)	86.6 (84.4–88.7)	81.5 (79.0–84.0)	79.9 (77.4–82.5)	90.1 (86.7–93.4)
Discordant^a^	79 vs 64	95 vs 105	95 vs 105	19 vs 14
OR (95% CI)	1.23 (0.9–1.7)	0.90 (0.7–1.2)	0.79 (0.6–1.0)	1.4 (0.6–2.9)
**Normal Cytology –*****N*****=2975**				
Agreement (%)	96.0	94.3	94.1	99.2
Kappa (95%CI)	81.4 (78.2–84.6)	73.9 (70.2–77.7)	72.6 (68.8–76.5)	83.6 (77.1–90.0)
Discordant^a^	66 vs 54	88 vs 81	85 vs 90	16 vs 8
OR (95% CI)	1.22 (0.8–1.8)	1.09 (0.8–1.5)	0.94 (0.7–1.3)	2.0 (0.8–5.4)
**All abnormal cytology –*****N*****=433**				
Agreement (%)	94.7	92.8	90.8	97.92
Kappa (95%CI)	87.9 (83.1–92.7)	83.0 (77.3–88.8)	78.3 (71.9–84.6)	94.2 (0.9–1.0)
Discordant^a^	13 vs 10	7 vs 24	10 vs 30	3 vs 6
OR (95% CI)	1.30 (0.5–3.3)	0.292 (0.1–0.7)	0.33 (1.5–0.7)	0.5 (0.1–2.3)
**Low grade cytology (borderline and mild) –*****N*****=370**				
Agreement (%)	94.3	92.8	89.7	98.1
Kappa (95%CI)	87.9 (83.9–92.9)	83.0 (77.3–88.8)	77.6 (70.9–84.3)	93.7 (89.1–98.3)
Discordant^a^	11 vs 10	7 vs 24	10 vs 28	1 vs 6
OR (95% CI)	1.1 (0.4–2.9)	0.29 (0.1–0.7)	0.36 (0.2–0.8)	0.2 (0–1.4)
**High grade cytology (CIN2+) –*****N*****=63**				
Agreement (%)	96.8	100	96.8	96.8
Discordant^a^	2 vs 0	0 vs 0	0 vs 2	2 vs 0
**Histology (CIN2+) –*****N*****=79**				
Agreement (%)	98.7	100	100	96.2
Discordant^a^	1 vs 0	0 vs 0	0 vs 1	2 vs 1
**Histology (CIN3+) –*****N*****=47**				
Agreement (%)	97.9	–	97.9	95.7
Discordant^a^	1 vs 0	0 vs 0	0 vs 1	2 vs 0

a Xpert positive/cobas negative vs Xpert negative/cobas positive.

**Table E1:** Sensitivity, positive predictive value and negative predictive value for CIN2+ and CIN3+ and specificity for <CIN2 and <CIN3.

**HPV test**	**Sensitivity (95%CI)**	**Specificity (95%CI)**	**PPV (95% CI)**
**Age**			
**CIN2+ (*****N*****=79)**			
**Xpert**			
All	98.7 (93.1–99.8)	82.3 (81.0–83. 6)	11.7 (9.3–14.4)
20–29	100 (93.1–100)	63.4 (59.9–66.8)	15.5 (11.8–19.8)
30–60	96.3 (81.0–99.4)	89.3 (88.1–90.4)	7.8 (5.2–11.3)
**COBAS**			
All	97.5 (91.1–99.6)	82.7 (81.4–84.0)	11.8 (9.4–14.5)
20–29	98.1 (89.7–99.68)	63.2 (59.7–66.6)	15.2 (11.5–19.5)
30–60	96.3 (81.0–99.4)	88.6 (87.3–89.8)	8.2 (5.4–11.8)
**hc2**			
All	98.7 (93.1–99.8)	82.3 (80.9–83.6)	11.7 (9.3–14.4)
20–29	100 (93.1–100)	65.3 (61.8–68.6)	16.2 (12.3–20.7)
30–60	96.3 (81.0–99.4)	87.0 (85.7–88.3)	7.3 (4.8–10.5)
**CIN3+ (*****N*****=47)**			
**Xpert**			
All	100 (92.4–100)	81.52 (80.2–82.8)	7.0 (5.2–9.3)
20–29	100 (89.3–100)	61.8 (58.4–65.2)	9.8 (6.9–13.5)
30–60	100 (76.7–100)	87.6 (86.3–88.9)	4.2 (2.3–7.0)
**COBAS**			
All	97.9 (88.7–99.6)	81.9 (80.6–83.2)	7.0 (5.2–9.3)
20–29	97.0 (84.2–99.5)	61.7 (58.2–65.1)	9.5 (6.6–13.2)
30–60	100 (76.7–100)	88.2 (86.9–89.4)	4.4 (2.4–7.3)
**hc2**			
All	100 (92.4–100)	81.2 (79.9–82.5)	6.9 (5.1–9.1)
20–29	100 (89.3–100)	63.7 (60.3–67.1)	10.3 (7.2–14.1)
30–60	100 (76.7–100)	86.6 (85.3–87.9)	3.9 (2.2–6.5)

## Appendix A

See Fig. A1.

## Appendix B

See Table B1.

## Appendix C

See Table C1.

## Appendix D

See Table D1.

## Appendix E

See Table E1.

## Footnotes

Appendix F Supplementary data associated with this article can be found in the online version at 10.1016/j.pvr.2015.05.002.
