# Supplementary material for: Performance of the Xpert HPV assay in women attending for cervical screening
Source: Papillomavirus Res. 2015 Jun 16;1:32–7. doi: 10.1016/j.pvr.2015.05.002 (PMC5886845; doi:10.1016/j.pvr.2015.05.002)
Supplement: Supplementary file 1 — Supplementary Material [file mmc1.pdf]

Appendix F Supplementary Table 1: HPV positivity by test, centre, cytology and histology result

**Centre/ HPV Test**	**Cytology** n (% of all with same cytology and centre)				**Histology** n (% of all with same histology and centre)	
	Normal	Low grade	High grade	All	CIN2+	CIN3+
Bristol	1120	18	9	1147	10	7
Edinburgh	911	173	48	1132	53	37
London	944	179	6	1129	16	3
All	2975	370	63	3408	79	47
**Xpert +ve**						
Bristol	121 (10.8)	12 (66.7)	9 (100)	142 (12.4)	10 (100)	7 (100)
Edinburgh	116 (12.7)	121 (69.9)	48 (100)	285 (25.2)	52 (98.1)	37 (100)
London	137 (14.5)	98 (54.7)	6 (100)	241 (21.3)	16 (100)	3 (100)
All	374 (12.6)	231 (62.4)	63 (100)	668 (19.6)	78 (98.7)	47 (100)
**Cobas +ve**						
Bristol	111 (9.9)	12 (66.7)	9 (100)	132 (11.5)	10 (100)	7 (100)
Edinburgh	116 (12.7)	123 (71.1)	47 (97.9)	286 (25.3)	52 (98.1)	37 (100)
London	135 (14.3)	95 (53.1)	5 (83.3)	235 (20.8)	15 (93.4)	2 (66.7)
All	362 (12.2)	230 (62.2)	61 (96.8)	653 (19.2)	77 (97.5)	46 (97.9)
**hc2 +ve**						
Bristol	118 (10.5)	13 (72.2)	9 (100)	140 (12.2)	10 (100)	7 (100)
Edinburgh	117 (12.8)	127 (73.4)	48 (100)	292 (25.8)	52 (98.1)	37 (100)
London	132 (13.9)	108 (60.3)	6 (100)	246 (21.8)	16 (100)	3 (100)
All	367 (12.3)	248 (67.0)	63 (100)	678 (19.9)	78 (98.7)	47 (100)

Appendix G Supplementary Table 2: Sensitivity, specificity and PPV of a positive HPV test for CIN2+ and CIN3+ by centre and test

Histology<br>HPV test	Sensitivity (95%CI)	Specificity (95%CI)	PPV (95% CI)
**CIN2+**			
**All**			
Xpert	98.7 (93.2-100)	82.3 (80.9-83.6)	11.7 (9.3-14.4)
Cobas	97.5 (91.1-99.6)	82.7 (81.4-84.0)	11.8 (9.4-14.4)
hc2	98.7 (93.2-100)	82.0 (80.6-83.3)	11.5 (9.2-14.2)
**Bristol**			
Xpert	100 (69.2-100)	88.4 (86.4-90.2)	7.0 (3.4-12.6)
Cobas	100 (69.2-100)	89.3 (87.3-91.0)	7.6 (3.7-13.5)
hc2	100 (69.2-100)	88.6 (86.6-90.4)	7.1 (3.5-12.7)
**Edinburgh**			
Xpert	98.1 (89.9-100)	78.4 (75.8-80.8)	18.3 (13.9-23.2)
Cobas	98.1 (89.93-100)	78.3 (75.7-80.7)	18.2 (13.9-23.2)
hc2	98.1 (89.93-100)	77.8 (75.2-80.2)	17.8 (13.6-22.7)
**London**			
Xpert	100 (79.4-100)	79.8 (77.3-82.1)	6.6 (3.8-10.6)
Cobas	93.8 (69.8-99.8)	80.2 (77.8-82.5)	6.4 (3.6-10.3)
hc2	100 (79.4-100)	79.3 (76.8-81.7)	6.5 (3.8-10.4)
**CIN3+**			
**All**			
Xpert	100 (92.4-100)	81.5 (80.2-82.8)	7.0 (5.2–9.3)
Cobas	97.9 (88.7-99.6)	81.9 (80.6-83.2)	7.0 (5.2-9.3)
hc2	100 (92.4-100)	81.2 (79.9-82.5)	6.9 (5.1-9.1)
**Bristol**			
Xpert	100 (59.0-100)	88.2 (86.1-90.0)	4.9 (2.0-9.9)
Cobas	100 (59.0-100)	89.0 (87.1-90.1)	5.3 (2.2-10.6)
hc2	100 (59.0-100)	88.3 (86.3-90.1)	5.0 (2.0-10.0)
**Edinburgh**			
Xpert	100 (90.5-100)	77.4 (74.8-79.8)	13.0 (9.3-17.5)
Cobas	100 (90.5-100)	77.3 (74.7-79.7)	12.9 (9.3-17.4)
hc2	100 (90.51100)	76.7 (74.1-79.2)	12.7 (9.1-17.0)
**London**			
Xpert	100 (29.2-100)	78.9 (76.4-81.2)	1.2 (0.3-3.6)
Cobas	66.7 (9.4-9.2)	79.4 (76.9-81.7)	0.9 (0.1-3.0)
hc2	100 (29.2-100)	78.4 (75.9-80.8)	1.2 (0.3-3.5)
